# Effects of a spatially heterogeneous nutrient distribution on the growth of clonal wetland plants

**DOI:** 10.1186/s12898-020-00327-1

**Published:** 2020-11-13

**Authors:** Hongwei Yu, Ligong Wang, Chunhua Liu, Dan Yu, Jiuhui Qu

**Affiliations:** 1 The National Field Station of Freshwater Ecosystem of Liangzi Lake, Department of Ecology, College of Life Sciences, Wuhan University, Wuhan, 430072 China; 2 Center for Water and Ecology, State Key Joint Laboratory of Environment Simulation and Pollution Control, School of Environment, Tsinghua University, Beijing, 100084 China; 3 Research Center for Eco-Environmental Sciences, Chinese Academy of Sciences, Beijing, China

**Keywords:** Clonal wetland plants, Guerrilla growth form, Phalanx growth form, Heterogeneity, Homogeneity

## Abstract

**Background:**

Clonal plants are important in maintaining wetland ecosystems. The main growth types of clonal plants are the guerrilla and phalanx types. However, little is known about the effects of these different clonal growth types on plant plasticity in response to heterogeneous resource distribution. We compared the growth performance of clonal wetland plants exhibiting the two growth forms (guerrilla growth form: *Scirpus yagara*, *Typha orientalis*, *Phragmites australis* and *Sparganium stoloniferum*; phalanx growth form: *Acorus calamus*, *Schoenoplectus tabernaemontani* and *Butomus umbellatus*) grown in soil substrates that were either homogeneous or heterogeneous but had the same total amount of nutrients.

**Results:**

We found that the morphological traits (plant height, ramet number, spacer diameter and length) and biomass accumulation of the guerrilla clonal plants (*T. orientalis*) were significantly enhanced by heterogeneity, but those of the phalanx clonal plants (*A. calamus*, *S. tabernaemontani* and *B. umbellatus*) were not. The results showed that the benefits of environmental heterogeneity to clonal plants may be correlated with the type of clonal structure.

**Conclusions:**

Guerrilla clonal plants, which have a dispersed, flexible linear structure, are better suited to habitats with heterogeneous resources. Phalanx clonal plants, which form compact structures, are better suited to habitats with homogeneous resources. Thus, wetland clonal species with the guerrilla clonal structure benefit more from soil nutrient heterogeneity.

## Background

Wetlands are unique areas that have characteristics of both land and water ecosystems and are among the world’s most productive environments [30]. Clonal growth forms dominate in many major biomes worldwide and are successful in wetland plant communities; thus, clonal plants play important roles in maintaining wetland ecosystems [25, 29, 31]. For example, clonal plants cover 66.69% of wetlands in China [31]. Emergent macrophytes have been shown to play an important role in wetland ecosystems [9, 37]. In aquatic habitats, vegetative propagation predominates among plant taxa; for instance, the majority of wetland species are rhizomatous clonal plants [32]. Clonal plants have special clonal life-history traits: I. trade-offs between clonal growth and reproduction [14]; II. clonal growth forms [34]; III. clonal plasticity [35]; and IV. clonal integration [2, 46]. For example, clonal plants can share resources (nutrients, water, etc.) among individual units through clonal integration, which increases plant survival and growth performance in habitats with different patterns of resource availability. In addition, clonal plants can increase their viability through risk sharing and resource storage [15, 44]. Thus, clonal plants have a strong ability to adapt to environmental pressure and resist disturbance.

Plants in natural habitats often experience heterogeneity in the spatial and temporal distribution of soil nutrients [21, 46]. Wetland plants, especially emergent macrophytes, are sensitive to the distribution of soil pollutants and nutrients during their growth process [7, 45]. In addition, clonal plants often exhibit more sensitive reactions than non-clonal plants; for example, the ramets of clonal plants interconnected by spacers (rhizomes, stolons, etc.) are often located in high-quality patches to efficiently utilize heterogeneously distributed resources [1, 12]. Spatial heterogeneity in soil nutrient availability can affect the growth performance of individual plants and the productivity and structure of plant communities [23, 36, 45]. For example, changing the spatial scale of nutrient heterogeneity can change the relative richness of species grown in mixtures [33, 43]. Thus, the heterogeneous distribution of soil nutrients may affect the fragile ecological stability of wetlands. Investigating the relationship between heterogeneous soil nutrient distribution and clonal wetland plants is highly important for shedding more light on the mechanisms of vegetation restoration.

Clonal plants with different clonal structure types may exhibit different strategies for adapting to their habitats and have different capacities for horizontal spreading [34, 42]. The main types of clonal plant growth are the guerrilla and phalanx types, but there are also many intermediate types [31, 34]. Clonal wetland plants that exhibit the phalanx structure, which consists of highly aggregated ramets connected by few and/or short spacers [24, 39]. In contrast, clonal wetland plants that exhibit the guerrilla growth form have a flexible distribution of ramets connected by many and/or long spacers [24, 39]. Phalanx clonal plants thrive in stable and homogeneous habitats, while guerrilla clonal plants grow in disturbed and heterogeneous habitats [41, 28, 40]. For example, soil nutrient heterogeneity significantly increased the relative yield of the guerrilla clonal plant *Bolboschoenus planiculmis* and decreased that of the phalanx clonal plant *Carex neurocarpa* [39]. However, few studies have focused on how clonal growth forms affect the responses of wetland plants to resource heterogeneity.

We designed an experiment to address the growth performance of clonal wetland plants in an environment of soil resource heterogeneity. The following questions were addressed: (1) Does soil nutrient heterogeneity have significant effects on the growth performance of clonal wetland plants? and (2) how do the growth responses of plants with different clonal growth forms to heterogeneous soil nutrients differ?

## Materials and methods

### Study area

The present study was conducted in Arongqi County, Inner Mongolia, China (48°10.883′ N, 123° 22.699′ E; altitude: 206 m). The Alun River in Arongqi County is a perennial flowing water body. The average annual temperature is 16.9 °C, the average annual precipitation is 470–570 mm, the average annual evaporation is 1400–1600 mm, and the average annual sunshine duration is 2600–2700 h in region.

### Plant materials

*Scirpus yagara*, *Typha orientalis*, *Phragmites australis*, *Sparganium stoloniferum*, *Acorus calamus*, *Schoenoplectus tabernaemontani* and *Butomus umbellatus* are emergent-rooted wetland plants. These seven species, which commonly co-occur in many freshwater ecosystems and are the dominant species in various wetland habitats, were selected for this study. These plants have obvious clonal growth structures and can therefore be used to effectively compare the response mechanisms of the two clonal structures to heterogeneity.

#### Guerrilla clonal plants

**Fig. 1:**
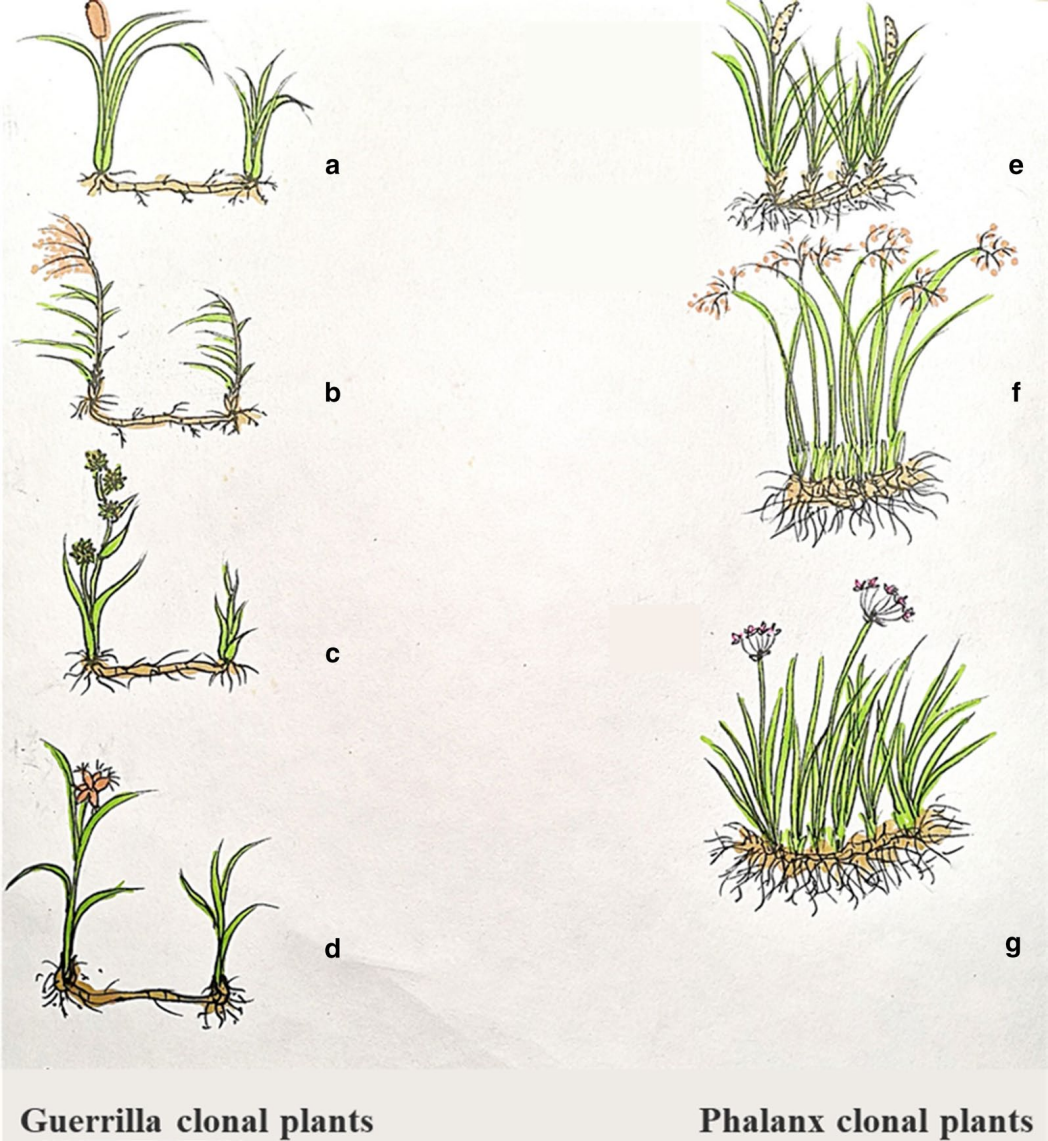
Plant Materials. Guerrilla clonal plants: **a** *Typha orientalis*; **b** *Phragmites australis*; **c** *Sparganium stoloniferum*; **d** *Scirpus yagara*. Phalanx clonal plants: **e** *Acorus calamus*; **f** *Schoenoplectus tabernaemontani*; **g** *Butomus umbellatus*

*Scirpus yagara*, *Typha orientalis*, *Phragmites australis*, *Sparganium stoloniferum* are perennial, rhizomatous, herbaceous clonal plants that show the guerrilla growth form (Fig. 1). These plants can produce isolated ramets via long spacers, resulting in widely spaced ramets called “spreading ramets” [6, 24, 41].

#### Phalanx clonal plants

*Acorus calamus*, *Schoenoplectus tabernaemontani* and *Butomus umbellatus* are perennial, rhizomatous, herbaceous clonal plants that show the phalanx growth form (Fig. 1). These plants grow few short spacers, resulting in closely packed ramets called “clumping ramets” [4, 6, 20].

### Experimental design

On April 12, 2016, ramets from each of the seven species were collected from the riparian zone of the Alun River. All of the collected ramets were precultivated in plastic buckets (70 cm long × 50 cm wide x 47 cm deep) with 20 cm of Alun River sediment (soil: mean ± SE, 0.29 ± 0.03 mg.g^−1^ N; 0.53 ± 0.02 mg.g^−1^ P; 31.64 ± 1.12 mg.g^−1^ organic material content) and 5 cm of Alun River water (water: 0.86 ± 0.14 mg.L^−1^N; 0.16 ± 0.04 mg.L^−1^ P) for approximately 60 days in the greenhouse. After culturing, 26 morphologically identical rooted ramets from each species (height: approximately 30 cm for *S. tabernaemontani*, *T. orientalis*, *P. australis* and *A. calamus*; approximately 20 cm for *S. stoloniferum*, *B. yagara*, and *B. umbellatus*) were selected. Ten ramets of each species were randomly selected to measure their initial dry biomass.

**Fig. 2:**
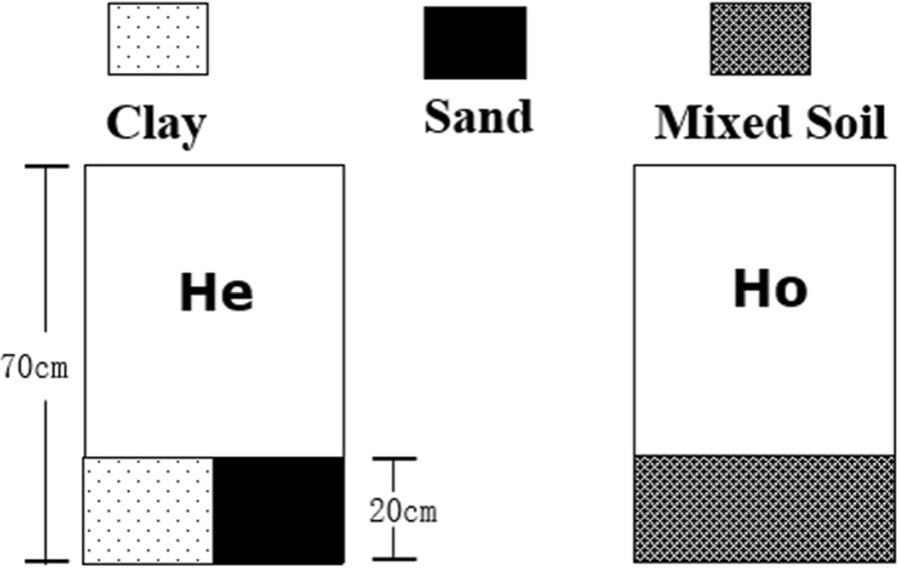
Schematic representation of soil substrate types. The light grey area in **He** was filled with lake sediment, and the dotted area was filled with sand. The shaded area in **Ho** represents an even mixture of the same amount of lake sediment and sand

This experiment was set up in buckets (70 cm diameter x 70 cm height) with two substrate types: I. the heterogeneous soil treatment (HE), in which the buckets were divided into two areas (Fig. 2); one area was filled with river clay (soil: mean ± SE, 0.32 ± 0.02 mg.g^−1^ N; 0.55 ± 0.02 mg.g^−1^ P; 32.57 ± 1.21 mg.g^−1^ organic material content), and the other area was filled with pure sand; II. The homogeneous soil treatment (HO) was a mixture of the same total amount of river clay and sand per bucket. The total amount of soil nutrients was the same in all treatments. On July 2, 2016, 8 ramets of each species were planted at the intersection of the clay and sand areas of the heterogeneous buckets to ensure equal access to high-nutrient and low-nutrient patches. The remaining 8 ramets of each species were planted at the centre of the homogeneous soil buckets. One plant of each species was planted in each bucket. Each treatment was replicated eight times, and 112 buckets were used in total. Each treatment was watered with purified water every 2–3d to minimize the limitation of their growth due to water availability. All plant materials were harvested on September 22, 2016, the total number of ramets per plant was recorded, and the plant height, spacer length and diameter were measured. Each plant was then divided into its aboveground (leaves and stems above the soil surface) and belowground parts (roots and rhizomes), dried at 70 °C for 5d and weighed. The belowground/aboveground biomass ratio was calculated as follows:

1 $$\frac{{{\text{Below}}\;{\text{ground}}}}{{{\text{Above}}\;{\text{ground}}}} \;{\text{ratio}} \;(\text{g}\, \text{g}^{-1}) = \frac{{ {\text{Root mass}} + {\text{Rhizome mass }}}}{{{\text{Leaf mass}} + {\text{Stem mass}}}}.$$

### Statistical analysis

When necessary, the data were transformed and normalized. Thus, all data on plant traits met the assumptions of normality and homogeneity of variance prior to analysis. One-way ANOVA was applied to test the effects of the soil treatment on plant traits. Growth traits were analysed using a three-way nested ANOVA with soil nutrient heterogeneity (homogeneous vs. heterogeneous), growth form (phalanx vs. guerrilla), and species nested within growth forms as factors. All data analyses were performed using SPSS 22.0 (SPSS, Chicago, IL, United States).

## Results

Soil nutrient heterogeneity had significant effects on the ramet number and belowground/aboveground biomass ratio, while clonal growth form did not significantly affect aboveground biomass or spacer length (Table 1). Soil nutrient heterogeneity *x* growth form had significant effects on the biomass and morphological characteristics, except spacer diameter, of the seven species (Table 1).

**Table 1:** Results of the three-way nested ANOVA examining the effects of soil nutrient heterogeneity (homogeneous vs. heterogeneous), growth form (phalanx vs. guerrilla), species (nested within growth forms) and their interaction on growth traits

	Effect				
	H	G	S-G	H x G	H x (S-G)
Aboveground biomass (g)	0.333^ns^	0.049^ns^	64.658^***^	29.223^***^	4.541^**^
Belowground biomass (g)	2.790^ns^	25.248^***^	81.580^***^	29.653^***^	3.079^*^
Total biomass (g)	0.403^ns^	15.078^***^	91.644^***^	38.097^***^	3.199^*^
Plant height (cm)	1.182^ns^	4.780^*^	326.880^***^	73.725^***^	8.180^***^
Ramet number	13.341^***^	11.985^***^	101.228^***^	63.315^***^	3.297^**^
Belowground/aboveground ratio (g.g^−1^)	5.782^*^	55.707^***^	37.146^***^	4.460^*^	2.648^*^
Spacer diameter (mm)	0.001^ns^	222.237^***^	67.196^***^	3.648^ns^	1.181^ns^
Spacer length (cm)	1.689^ns^	0.007^ns^	20.190^***^	38.891^***^	3.037^*^

*Soil nutrient heterogeneity (H), growth form (G) and species (S-G). * Values are *F*; significant *P* values (*P < 0.05, **P < 0.01, ***P < 0.001 and ^ns^*P *≥ 0.05)

### Guerrilla clonal plants

**Fig. 3:**
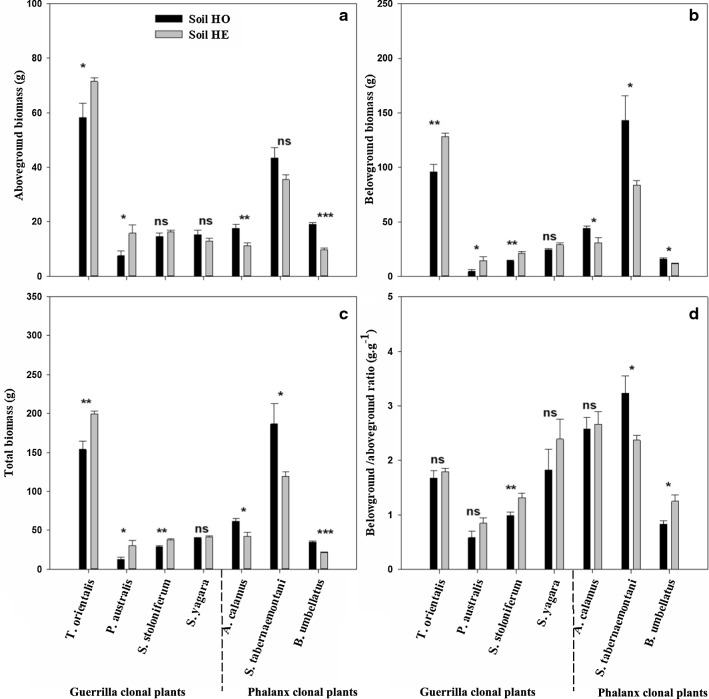
Effects of substrate heterogeneity on biomass accumulation and distribution in seven clonal plants. Values are **a** aboveground biomass, **b** belowground biomass, **c** total biomass, and **d** belowground/aboveground biomass ratio. Values are means ± SEs. The bars with different lowercase letters are significantly different

**Fig. 4:**
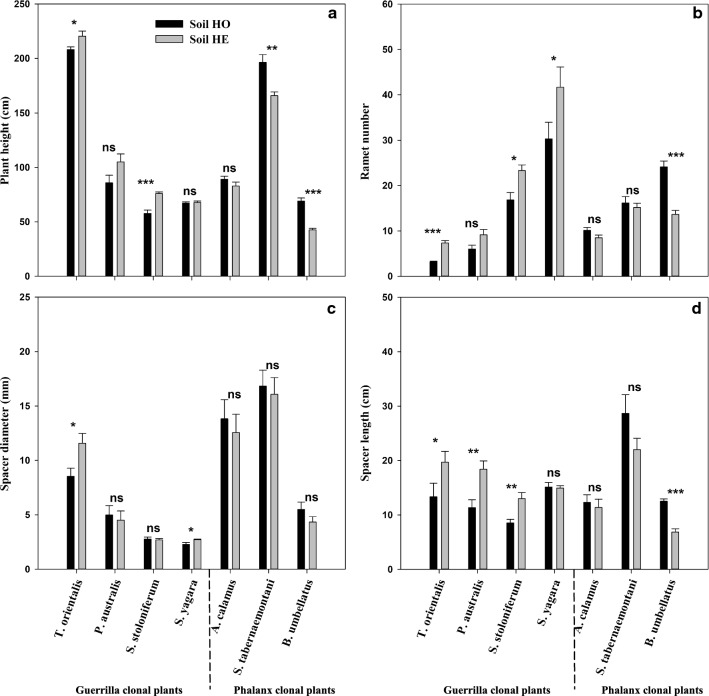
Effects of substrate heterogeneity on the morphological traits of seven clonal plants. Values are **a** plant height, **b** ramet number, **c** spacer diameter, and **d** spacer length. Values are means ± SEs. The bars with different lowercase letters are significantly different

The heterogeneous soil treatment had a positive impact on the growth performance of guerrilla clonal plants. For example, the heterogeneous soil treatment significantly increased the biomass and morphological characteristics, except the belowground/aboveground biomass ratio, of *T. orientalis* (Figs. 3 and 4). Significantly higher biomass and spacer length were observed in *P. australis* in the heterogeneous soil treatment (Figs. 3 and 4). Additionally, the heterogeneous soil treatment significantly affected biomass accumulation and allocation, and increased some morphological traits (plant height, ramet number, spacer length) in guerrilla clonal plants. However, for *S. yagara*, a significantly higher ramet number and spacer diameter were observed in the heterogeneous soil treatment (Fig. 4).

### Phalanx clonal plants

The heterogeneous soil treatment had a negative impact on the growth performance of the phalanx clonal plants. For example, significantly lower biomass of *A. calamus* was observed in the heterogeneous soil treatment, but no effect was observed on its morphological characteristics (Figs. 3 and 4). The heterogeneous soil treatment significantly increased the belowground biomass, total biomass, belowground/aboveground biomass ratio and plant height of *S. tabernaemontani* (Figs. 3 and 4). The heterogeneous soil treatment significantly decreased the growth traits, except the spacer diameter, of *B. umbellatus* (Figs. 3 and 4).

## Discussion

A few experimental results have shown that resource heterogeneity can increase plant performance, as measured by the accumulation and allocation of biomass. This has been observed at the levels of individual plants, populations and whole communities [3, 16 and 22]. However, other experiments have demonstrated that the positive responses of clonal plants to resource heterogeneity may not always be adaptive or may be temporary [10, 17, 27 and 45]. In this study, the performance of clonal wetland plants in heterogeneous environments was correlated with the type of clonal growth forms. For example, the heterogeneous distribution of nutrients in the soil substrate significantly increased the growth performance of the guerrilla clonal plants (*T. orientalis*; Figs. 3 and 4), but the homogeneous distribution of nutrients in the soil substrate significantly increased the growth performance of the phalanx clonal plants (*A. calamus, S. tabernaemontani* and *B. umbellatus*; Figs. 3 and 4). This may be because guerrilla clonal plants can spread rapidly and produce offspring vegetatively by forming flexible, spreading offspring ramets, which have access to dispersed resources. Phalanx clonal plants usually spread slowly and form aggregated clones, which have access to centralized resources [8, 29, 31]. In addition, some species exhibit a trade-off between the two growth forms in different habits and successional stages. For instance, *Leymus secalinus* can respond and adapt to small-scale heterogeneity in its resource supply by altering the plasticity of its spacer morphology [41]. Therefore, guerrilla clonal plants may be better suited to a heterogeneous distribution of soil nutrients, and phalanx clonal plants show significantly enhanced growth performance in homogeneous soil nutrient conditions.

Owing to their strong horizontal expansion ability and morphological plasticity, guerrilla clonal plants respond to nutrient conditions by concentrating their foraging organs (such as rhizomes, stolons, and corms) where nutrient levels are relatively high [12]. For example, *T. orientalis* produced more ramets and larger spacer diameters and lengths in the heterogeneous soil treatment (Fig. 4). In addition, guerrilla clonal plants can alter the branching angle and distribution of ramets as well as their biomass allocation ratio to obtain available resources [18, 38]. These specific clonal growth organs are characterized by different functional traits (such as dispersal, resource acquisition, storage, shoot cycling and protection) [32] and can adapt to diverse environments. Thus, clonal plants, especially guerrilla clonal plants, are widely used in the ecological remediation of various habitats.

Clonal plants often appear as pioneer species in the initial stages of community succession [26]. In the process of vegetation restoration, clonal plants play a dominant role in changing the vegetation community environment and maintaining community ecological function [11]. For example, *Psammochloa villosa* (a guerrilla clonal plant) can improve vegetation coverage and sand fixation in mobile dune patches [13]. Particle size is an important feature of soil, and it can greatly affect the growth of clonal plants [19]. In this experiment, the heterogeneous distribution of soil nutrients and the particle size may have inhibited the foraging behaviour of the phalanx clonal plants. This may have occurred because the mechanical resistance of the soil particles decelerated root growth and expansion [5].

Resources (light, water, nutrients, etc.) and environmental conditions (disturbances, geography, herbivory, etc.) exhibit spatial and temporal heterogeneity, which is ubiquitous within natural habitats [21, 45]. In this study, *T. orientalis* showed high ecological adaptability to heterogeneous resource habitats. In addition, the guerrilla clonal plants (*T. orientalis*, *P. australis* and *S. stoloniferum*) accumulated more belowground biomass in the heterogeneous soil, in which larger foraging organ masses may explore and occupy resource-rich patches (Fig. 1). These identical individuals can share resources and stress through the physical connections of their spacers [2, 42]. This is why the guerrilla growth is very common in early successional stages and disturbed habitats.

## Conclusions

In conclusion, the heterogeneity of soil nutrients promotes the growth of guerrilla clonal plants, especially that of *T. orientalis*. The flexible clonal structure of guerrilla clonal plants can be used to effectively utilize resources. Future studies should focus on how various ecological factors, such as temperature and competition, affect growth responses to resource heterogeneity.

## Data Availability

The data are available from the corresponding author upon reasonable request and with permission from Wuhan University.

## Abbreviations

*T. orientalis*: *Typha orientalis*
*A. calamus*: *Acorus calamus*
*S. tabernaemontani*: *Schoenoplectus tabernaemontani*
*B. umbellatus*: *Butomus umbellatus*
*S. yagara*: *Scirpus yagara*
*P. australis*: *Phragmites australis*
*S. stoloniferum*: *Sparganium stoloniferum*
HE: Heterogeneous soil treatment
HO: Homogeneous soil treatment
